# Patient engagement for quality improvement and knowledge translation in paediatric orthopaedic care – a patient partner led initiative

**DOI:** 10.1186/s41687-026-01062-9

**Published:** 2026-05-14

**Authors:** Marisa Wong, Philippa Crerar, Anthony Philip Cooper, Harpreet Chhina

**Affiliations:** 1 Faculty of Anatomy and Cell Biology, Western University, London, ON Canada; 2 Faculty of Health Science, University of Waterloo, Waterloo, ON Canada; 3 Department of Orthopaedics, BC Children’s Hospital, Vancouver, BC Canada; 4 Department of Orthopaedics, University of British Columbia, Vancouver, BC Canada

**Keywords:** Patient-oriented research, Patient-led, Focus groups, Patient collaboration, Knowledge translation, Patient-centric

## Abstract

**Background:**

In healthcare, a shift towards patient-centric models has gained traction, emphasizing patient empowerment. This is exemplified by initiatives such as the Canadian Institutes of Health Research (CIHR) Strategy for Patient-Oriented Research (SPOR), which aims to foster a more sustainable and equitable healthcare system, as well as the increased support provided by the Patient-Centered Outcomes Research Institute (PCORI), which supports research that provides useful and reliable healthcare information to users. Despite progress in various healthcare domains, patient-engagement initiatives remain underexplored in paediatric orthopaedic care, highlighting a gap in tailored engagement strategies for this population.

**Methods:**

This project took place within the department of orthopaedics at a provincial tertiary care children’s hospital. Utilizing patient-partner led focus groups as a collaborative approach, we implemented engagement strategies guided by SPOR principles and informed by patient and caregiver feedback. Small focus groups facilitated meaningful discussions and allowed participants to contribute valuable insights into research, treatment, and knowledge translation (KT).

**Results:**

The focus groups identified action items to enhance patient engagement and research collaboration. Initiatives included the development of *patient and family directories, patient resources, patient partnerships, patient spotlights on Instagram, and clinic gathering rooms.* Co-creating KT materials ensured relevance and accessibility, fostering cooperation between patients, caregivers, and healthcare professionals.

**Conclusions:**

This initiative marks a shift towards patient-centric paediatric orthopaedic care, emphasizing inclusivity, support, and collaboration. Patient-partners played a pivotal role in the success of the project, ensuring patient perspectives were central to the planning and execution of initiatives.

## Introduction

In recent years, an ideological shift has emerged in healthcare, signaling a departure from conventional models towards a patient-centric approach [1]. This philosophy places emphasis on empowering patients as informed end-users, recognizing them not only as recipients of care, but as active contributors to their treatment [2]. Departure from traditional models emphasizes that patients benefit from a comprehensive understanding of their conditions and treatment plans [3].

Since 2017, the Canadian Institutes of Health Research’s (CIHR) Strategy for Patient-Oriented Research (SPOR) has been progressing with the goal of establishing a healthcare system that is more sustainable and equitable [4]. In 2024, the Patient-Centered Outcomes Research Institute (PCORI) put forward over $4 billion to support comparative clinical effectiveness research that aims to compare benefits and harms of various treatments, strategies, and approaches to care [5]. This shift extends beyond just administrative changes, emphasizing its tangible impact observed across various healthcare settings in both management and practice [6]. Previous initiatives focusing on enhancing patient knowledge, yielded substantial improvements in patient outcomes, treatment adherence, and overall patient satisfaction [6, 7]. Across various healthcare domains, patient-engagement programs have resulted in informed decision-making and heightened patient satisfaction [7].

In paediatric orthopaedics there is a gap in implementing patient-engagement initiatives. Patient engagement in pediatric orthopaedics is uniquely challenging due to children’s developmental stages, caregiver involvement, and the complexity of musculoskeletal conditions. Our project was centered on patient engagement within paediatric orthopaedic care in a provincial tertiary care children’s hospital. The exploration and implementation of engagement strategies were prompted by both the transformative potential of patient-engagement initiatives and the interest in knowledge translation (KT) from our patient population [4, 7].

As SPOR indicates, patient engagement involves “meaningful and active collaboration in governance, priority setting, conducting research and knowledge translation” [4]. Focus group discussions are an effective way to implement a collaborative patient centered approach to research [4, 8, 9]. Reflecting on past patient engagement activities in our clinic we identified both positive and negative sentiments. While some patients expressed support for the research, others questioned the direct benefit of their participation. This early patient engagement initiative provided valuable insights into the perspectives of our patient families, revealing a desire to support research but a lack of understanding regarding the purpose and benefits of participating in research.

As our project evolved on these early lessons, the primary aim became creating more patient-engagement opportunities that directly addressed the concerns of our population. Through collaborative efforts, we sought to co-create resources in the form of KT materials that resonate with the needs of our patient families. The overarching aim of this engagement program was to establish a sustainable system for keeping patient families informed about our research and clinic activities as well as obtain valuable input and partnership. We also sought to build on partnerships with existing patient-partners to co-create these KT pieces.

## Materials and methods

**Fig. 1:**
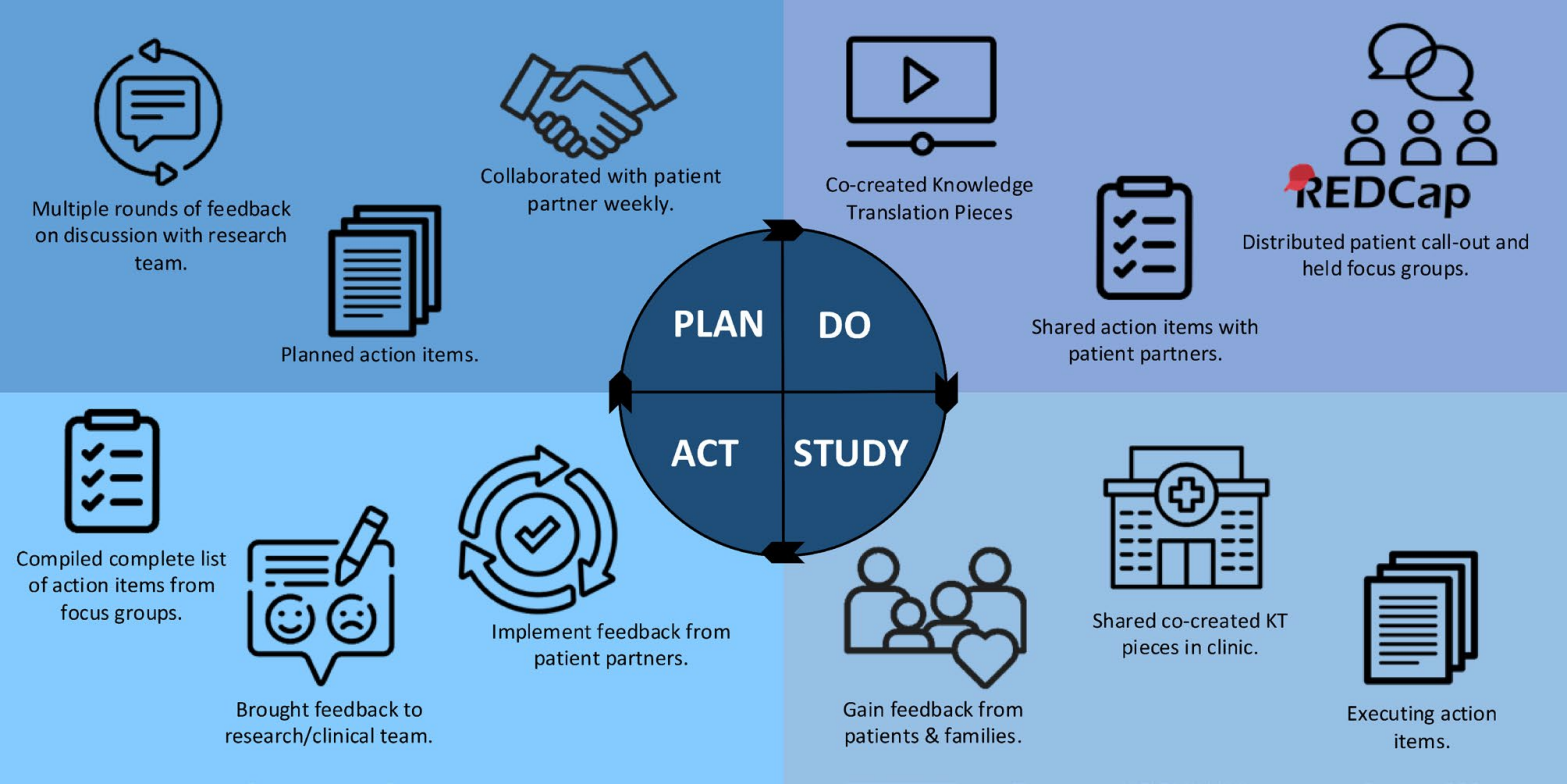
Quality improvement methodology using iterative plan–do–study–act (PDSA) cycles

This project was conducted as a patient-partner led quality improvement initiative within a paediatric orthopaedic clinic at a provincial tertiary care children’s hospital. The initiative was guided by the Strategy for Patient-Oriented Research (SPOR) Patient Engagement Framework and implemented using iterative Plan–Do–Study–Act (PDSA) cycles (Fig. 1) [10].

A youth patient-partner with lived experience receiving care in the clinic was engaged as a core member of the project team and led all patient-facing activities, including focus group facilitation. Patients and caregivers who had previously expressed interest in engagement were recruited via clinic handouts, direct email invitations, and social media outreach. An interest survey developed in Research Electronic Data Capture (REDCap) collected contact information, preferred meeting format, and availability [11]. Focus group participants consisted primarily of caregivers of paediatric patients aged 4–15 years receiving care of the hospital.

Focus groups explored patient and caregiver perspectives on research participation, quality-of-life (QOL) questionnaires, and preferences for knowledge translation (KT) materials. A research team member provided standardized explanations of orthopaedic registries and QOL data that were routinely collected in clinics, during the focus groups to support informed discussion. Following each focus group, structured debriefs were conducted with the patient-partner and research team to refine subsequent sessions in alignment with the PDSA framework.

Focus group outputs were synthesized using a rapid qualitative synthesis approach, with key ideas thematically grouped and translated into action items through consensus discussion among the research team. The finalized action items were validated through member-checking with the patient-partner, with select items reviewed by patient participants who initially raised them. While patient partners guided the prioritization, responsibility for implementation rests with the clinical and research team to ensure integration within existing clinic structures and to avoid placing undue burden on patients and families. Clinical and research team members routinely invite patients and parents to be research partners and share their experiences of diagnosis and treatment and co-create patient spotlights and knowledge translation materials. They are offered support and guidance as needed during the co-creation of patient spotlights and knowledge translation materials. Support from research team ensures sustainability of these initiatives.

## Outcomes and actions

Prior to this initiative, parent and patient-reported QOL surveys were routinely collected during clinic visits as part of ongoing research and registry activities; however, patients and caregivers received limited explanation regarding the purpose of these questionnaires, how the data were used, or how results informed clinical care. Information about research participation and QOL data collection was primarily conveyed verbally during clinic visits, with no standardized patient-facing materials or dedicated knowledge translation resources. This lack of transparency was identified by patients and caregivers as a barrier to meaningful engagement and informed participation.

Inspired by the positive outcomes of patient-centered approaches to care and research, we were called to action [1, 3, 6, 7, 12, 13]. We aimed to empower young patients through the patient-centric approaches and involve them and their caregivers in the processes of improving paediatric orthopaedic care and research.

Table 1 summarizes the action items that emerged directly from patient and caregiver focus groups conducted as part of the PDSA cycles. Action items reflect priorities identified by participants, with goals defined using participants’ own explanations of why each initiative would be meaningful or beneficial to them. For each action item, the prior state is documented alongside the current status to enable direct comparison and demonstrate accountability and impact over time. Action items were synthesized by the research team and subsequently validated through member-checking with a patient partner; select items were also reviewed with patient partners who initially raised the ideas. Prioritization was guided by anticipated patient impact, feasibility within existing clinical structures, resource requirements, and alignment with institutional governance and ethical considerations. Identified roadblocks or constraints are noted to promote transparency regarding implementation status.

**Table 1:** Resulting action items

Action Item	Goal	Previous state	Current Status	Roadblocks/Constraints
Patient and Family Directory	To foster connection between patients and families with similar diagnosis’ and/or treatment experiences	Natural connections evolved in the clinic due to the nature of the waiting room	Institutional restrictions on creating directory with patient information	Privacy and information constraints
Patient Resources	Better organization of patient resources on the program website	Outdated information	IMPLEMENTED: Added Patient Resources tab to website	Continuous updates by research team in consultation with the clinicians
Patient Partnerships	Better organization of patient resources on the program website	Connecting patient families, who share similar experiences of diagnosis and treatment, by the clinical team	IMPLEMENTED: A patient partner, studying design and animation, was invited to review and provide feedback for Program Website	Continuous updates by research team
Patient Spotlights on Instagram	Share personal narratives from patients and caregivers and encouraging meaningful engagement with a broader audience [4]	Some stories shared inconsistently. 1–3x a year	IMPLEMENTED & CONTINUED: Stories shared on Instagram 1/month	
Clinic Gathering Room	Provide a welcoming space for families to access educational materials and engage with others in comparable situations	The only place to gather was in the clinic waiting room	HELD	Hospital space and administration
Co-Create Knowledge Translation Materials	Create relevant materials that enhance patient understanding in an approachable format	No previously co-created resources	IMPLEMENTED: All patient resources listed on the website and shared in the clinic and waiting room are co-created with the help of patient partners and patient input	Continuous updates by research team
Improved Holistic Clinical Care	Increased support for emotional and dietary health for patients and caregivers undergoing complex procedures	Only by referral by NP and MD when caught. Not standard care practice	IMPLEMENTED: Addition of Dietician and Social Worker to the clinical team	Clinic funding, HR. Hiring of new allied health professionals

Action items generated from focus group discussions were synthesized by the research team and patient-partner and prioritized through consensus based on anticipated impact, feasibility within existing clinic structures, resource requirements, and alignment with institutional governance and ethical considerations (Table 1). Initiatives addressing patient understanding of research activities and QOL questionnaires, clarity of patient-facing information, and opportunities for engagement and partnership were prioritized for early implementation, as these were identified as the most immediate gaps and were feasible to implement using existing platforms.

Initiatives requiring additional resources, institutional approval, or ethical approvals, such as the patient and family directory, clinic gathering room, and expanded holistic supports, were designated as under consideration. This staged approach reflects a balance between patient-identified priorities and practical constraints while supporting sustainable, patient-centred improvements in paediatric orthopaedic care.

Collaborating with a patient-partner played a crucial role in the planning, execution, and success of this project. We managed her comfort levels through constant communication while prioritizing her input and ideas [4]. She proved instrumental, leading to a remarkable level of success in translating the project to other patients.

These engagement initiatives demonstrate a transformative shift towards a patient-centric paradigm in paediatric orthopaedics. This change not only addresses specific concerns but also redefines the patient-provider relationship. The continuing commitment to patient engagement reflects the optimization of clinical care and a shift to greater involvement of patients in the research process. Honouring our commitment to continuous quality improvement, we hope to continue our work and create a sustainable feedback-loop that maintains open lines of communication with our patient families. This collaborative approach not only enriches our research but also establishes a mutually beneficial partnership with our patient-community. Ensuring easy access to research findings empowers patient families, breaks down barriers, and fosters active engagement. Jargon-free communication in KT enhances the understanding of research significance, promoting collaboration by establishing a shared understanding between researchers and patients. Overall, accessibility and comprehension of research findings are key to enthusiastic patient engagement; however, this requires active collaboration and dedication to meeting the diverse needs of patients and their families.

## Learnings and next steps

Moving forward, we are committed to sustaining open communication with our patient families, co-creating KT materials, and continuously enhancing patient experiences to drive ongoing improvement in orthopaedic clinical care. This project provides valuable information about patient and caregiver experiences with clinical care and research, creating systems to incorporate feedback, and improve quality of care. A project evaluating the implementation of PROMs in clinical care and research from clinician’s, patient’s and parent’s perspective is in progress at this time.

We aim to continue co-creating KT pieces with our patients and caregivers while implementing feedback mechanisms on existing tools to ensure future materials are tailored to our population. We work to cultivate relationships with additional patient-partners who are eager to contribute to the sustainability of these changes.

## Data Availability

Data sharing is not applicable to this article as no datasets were generated or analyzed during the current study.

## Abbreviations

KT: Knowledge Translation
SPOR: Strategy for Patient-Oriented Research
